# An anesthesia protocol for robust and repeatable measurement of behavioral visceromotor responses to colorectal distension in mice

**DOI:** 10.3389/fpain.2023.1202590

**Published:** 2023-05-26

**Authors:** Shaopeng Zhang, Longtu Chen, Bin Feng

**Affiliations:** Department of Biomedical Engineering, University of Connecticut, Storrs, CT, United States

**Keywords:** anesthesia, visceromotor response, colorectal distension, pain, irritable bowel syndrome

## Abstract

**Introduction:**

Visceral motor responses (VMR) to graded colorectal distension (CRD) have been extensively implemented to assess the level of visceral pain in awake rodents, which are inevitably confounded by movement artifacts and cannot be conveniently implemented to assess invasive neuromodulation protocols for treating visceral pain. In this report, we present an optimized protocol with prolonged urethane infusion that enables robust and repeatable recordings of VMR to CRD in mice under deep anesthesia, providing a two-hour window to objectively assess the efficacy of visceral pain management strategies.

**Methods:**

During all surgical procedures, C57BL/6 mice of both sexes (8–12 weeks, 25–35 g) were anesthetized with 2% isoflurane inhalation. An abdominal incision was made to allow Teflon-coated stainless steel wire electrodes to be sutured to the oblique abdominal musculature. A thin polyethylene catheter (Φ 0.2 mm) was placed intraperitoneally and externalized from the abdominal incision for delivering the prolonged urethane infusion. A cylindric plastic-film balloon (Φ 8 mm x 15 mm when distended) was inserted intra-anally, and its depth into the colorectum was precisely controlled by measuring the distance between the end of the balloon and the anus. Subsequently, the mouse was switched from isoflurane anesthesia to the new urethane anesthesia protocol, which consisted of a bout of infusion (0.6 g urethane per kg weight, g/kg) administered intraperitoneally via the catheter and continuous low-dose infusion throughout the experiment at 0.15–0.23 g per kg weight per hour (g/kg/h).

**Results:**

Using this new anesthesia protocol, we systematically investigated the significant impact of balloon depth into the colorectum on evoked VMR, which showed a progressive reduction with increased balloon insertion depth from the rectal region into the distal colonic region. Intracolonic TNBS treatment induced enhanced VMR to CRD of the colonic region (>10 mm from the anus) only in male mice, whereas colonic VMR was not significantly altered by TNBS in female mice.

**Discussion:**

Conducting VMR to CRD in anesthetized mice using the current protocol will enable future objective assessments of various invasive neuromodulatory strategies for alleviating visceral pain.

## 1. Introduction

Managing visceral pain associated with functional gastrointestinal disorders (FGIDs) is an unmet clinical need despite the prevalence of visceral pain (1), the negative impact on patient's quality of life (2), and the significant financial burden relevant to visceral pain (3). In particular, visceral pain is the cardinal complaint of patients with irritable bowel syndrome (IBS), one of the FGIDs featuring increased visceral hypersensitivity to normal bowel function in the absence of apparent organ damage or inflammations. The “functional” nature of IBS challenges the discovery of quantifiable biological markers (biomarkers) for the diagnosis of IBS (4); IBS is often a diagnosis of exclusion in general medical practice (5). Reliable and quantifiable biomarkers can greatly guide the development of preclinical animal models and facilitate the translation of preclinical discoveries to successful medical interventions in clinics (4). Among the limited list of biomarker candidates, visceral hypersensitivity assessed by functional distal colorectal distension (CRD) was reported to closely correlate with the diagnosis of IBS in patients (6, 7), and thus has been widely used as an objective metric in preclinical animal studies of visceral pain (8–11). Unlike the convenient patient-reported visual analog score (VAS) of pain in clinical assessment, objective quantification of visceral hypersensitivity in rodents relies on the pseudoaffective reflex of visceromotor responses (VMR) as measured usually by the electromyographic (EMG) activities of the abdominal oblique musculature (12), and to a lesser extent by visual inspection of abdominal activities (13) and intraluminal colonic pressure (14). VMR to CRD recorded in conscious rodents has been widely implemented to screening pharmacological targets for alleviating visceral pain, including the N-methyl-D-aspartate (NMDA) receptor blocker (15), kappa opioid receptor blockers (16), blocker of paracellular permeability and myosin light chain kinase inhibitor (17), cannabinoid receptor antagonists (18), guanylate cyclase-C agonists (19, 20), and neurokinin receptor antagonists (21).

Recording VMR from conscious rodents is unavoidably confounded by the voluntary and involuntary limb and torso movement, which generally produces artifacts in the EMG recordings with magnitude comparable to and even higher than the VMR signals. In addition, the assessment of invasive pain-managing schemes cannot be conveniently conducted on awaked animals, including implantable neurostimulators that are promising non-drug alternatives for treating chronic visceral pain (22). Thus, VMR recorded in anesthetized rodents is potentially an ideal test bench for assessing various neuromodulation schemes to treat IBS-related visceral pain. However, the VMR is a pseudoaffective reflex subserved by spino-bulbo-spinal neural circuitry. Therefore, most general anesthesia that suppresses neural activities in the pons of the brain stem usually inhibits VMR to CRD (12). In clear contrast, urethane has been proved to preserve the spino-bulbo-spinal reflex with a single injection and was extensively applied in recording micturition responses during slow bladder filling in rats (23) and mice (24). Also, several recent publications from us and others showed that VMR to CRD can be recorded from mice receiving a single i.p. injection of urethane (22, 25). In the current study, we focus on establishing a new urethane anesthesia protocol that enables repeated VMR recordings to CRD in mice under hours-long anesthesia. The new protocol consists of an initial bout of urethane infusion and a continuous low-dose i.p. urethane infusion via a catheter. We optimized the urethane infusion protocol to achieve robust and repeatable VMR recordings to CRD for up to 2 h. Since the distal colon and rectum are innervated by distinct afferent pathways (26), we systematically assessed the effect of balloon depth in the colorectum on the VMR recordings to CRD in both naive and visceral hypersensitive mice induced by intracolonic enema of 2,4,6-trinitrobenzenesulfonic acid (TNBS).

## 2. Methods

All experimental procedures were approved by the University of Connecticut Institutional Animal Care and Use Committee.

### 2.1. Surgical preparation and experiment setup

**Figure 1:**
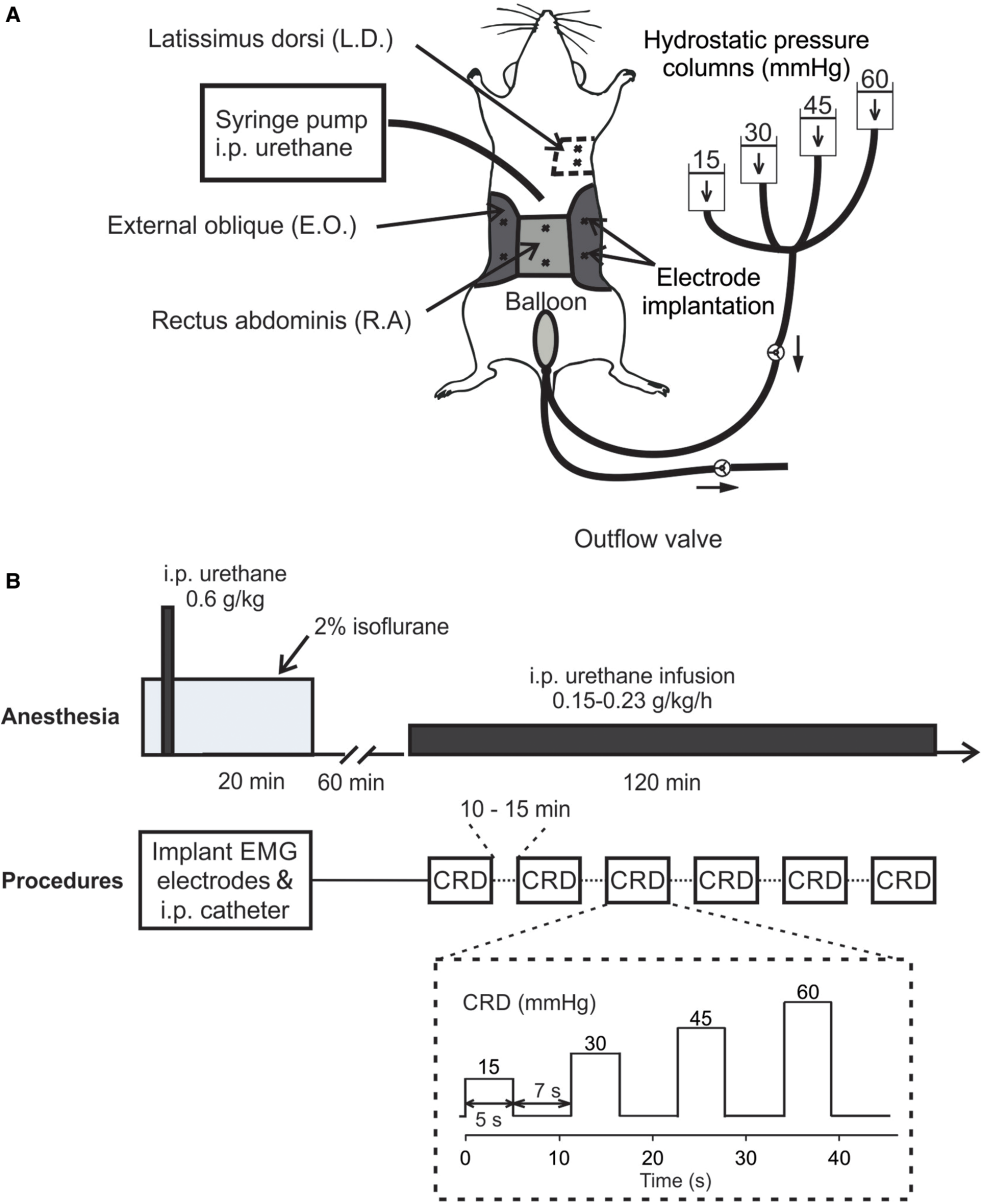
*In vivo* setup and procedure for measuring VMR to CRD using a new urethane anesthesia protocol. (**A**) A schematic representation of the experimental setup for measuring the EMG activities from abdominal muscles during CRD. The CRD was delivered using hydrostatic saline columns of 15, 30, 45, and 60 mmHg. (**B**) The anesthesia protocol involved an initial bout of urethane followed by a continuous low-dose urethane infusion. The electrode implantation and i.p. catheterization were performed under isoflurane anesthesia (2%), which was cleared for 60 min before the low-dose urethane infusion. Repeated CRD was conducted with 10–15 min intervals between successive CRDs. VMR, visceral motor responses; CRD, colorectal distension; EMG, electromyographic; i.p., intraperitoneal; E.O., external oblique; R.A., rectus abdominis; L.D., latissimus dorsi.

C57BL/6 mice of both sexes (8–12 weeks, 25–35 g) were anesthetized by 2% isoflurane inhalation during all surgical procedures to avoid spontaneous or volitional movement. Throughout the anesthesia phase, mice were placed on a feedback-controlled heating pad (HP-150, Auber Instruments, Alpharetta, GA, United States) to maintain consistent body temperature of ∼37°C. To record abdominal EMG, we used a pair of Teflon-coated stainless wires (Conner Wire, Chatworth, CA, United States), exposed the ∼0.5 mm tip, and sutured them directly to the muscle with hypoallergenic bioabsorbable sutures (polyglactin, Ethicon) with 1 mm separation between both electrodes. The other ends of the electrodes were connected to a differential amplifier (Model 1700, A-M Systems, Sequim, WA, United States) with the ground wire connected to the mouse tail. During surgery, three pairs of Teflon-coated stainless steel wire electrodes were sutured to the rectus abdominis (R.A.) and the external oblique (E.O.) musculature immediately above the inguinal ligament (both sides for E.O.) for measuring the EMG responses to CRD (as shown in Figure 1A). One pair of Teflon-coated stainless steel wire electrodes was sutured to the latissimus dorsi (L.D.) musculature for measuring respiratory rate (RR). In addition, a plastic catheter (Φ 0.2 mm) was placed intraperitoneally beneath the transversus abdominis musculature for the infusion of urethane. Incisions on muscle layers were closed by bioabsorbable sutures (polyglactin, Ethicon) and on the skin layer by nonabsorbable sutures (Vicryl, Ethicon). A flexible lubricated polyethylene-film balloon (Φ 8 mm × 15 mm) was inserted intra-anally into the precise location in the colorectum by measuring and maintaining the 5–30 mm distance between the end of the balloon and the anus (i.e., the balloon depth) via taping the connected plastic inflating catheter to the tail. The balloon was pressurized by a custom-built distension device consisting of four hydrostatic columns of water set at 15, 30, 45, and 60 mmHg pressures (22) (shown in Figure 1A). Computer-controlled solenoid valves were implemented to regulate the onset and termination of CRD, which as shown in Figure 1A consisted of four ascending pressure steps of 5 s duration and 7 s between successive steps (15, 30, 45, and 60 mmHg). EMG responses were recorded using a differential amplifier and a digitizer (CED 1401, Cambridge Electronic Design Limited, Cambridge, United Kingdom).

### 2.2. New urethane anesthesia protocol for VMR recordings

The new urethane anesthesia protocol consists of an initial bout of infusion right after the catheter insertion as described above and a continuous low-dose infusion of urethane 60 min after terminating isoflurane (shown in Figure 1B). First, the initial dose of 0.6 g/kg urethane was administered intraperitoneally via the catheter inserted during the above surgical preparation. Isoflurane inhalation is removed after completing all the surgical procedures followed by another 60-min clearing period. Then, we started the continuous i.p. infusion of low-dose urethane [0.15–0.23 g per kg weight per hour (g/kg/h)] through the catheter to maintain systemic anesthesia for 2 h. During the 2-h window, VMR to CRD were assessed six times with 10–15 min between successive tests.

**Figure 2:**
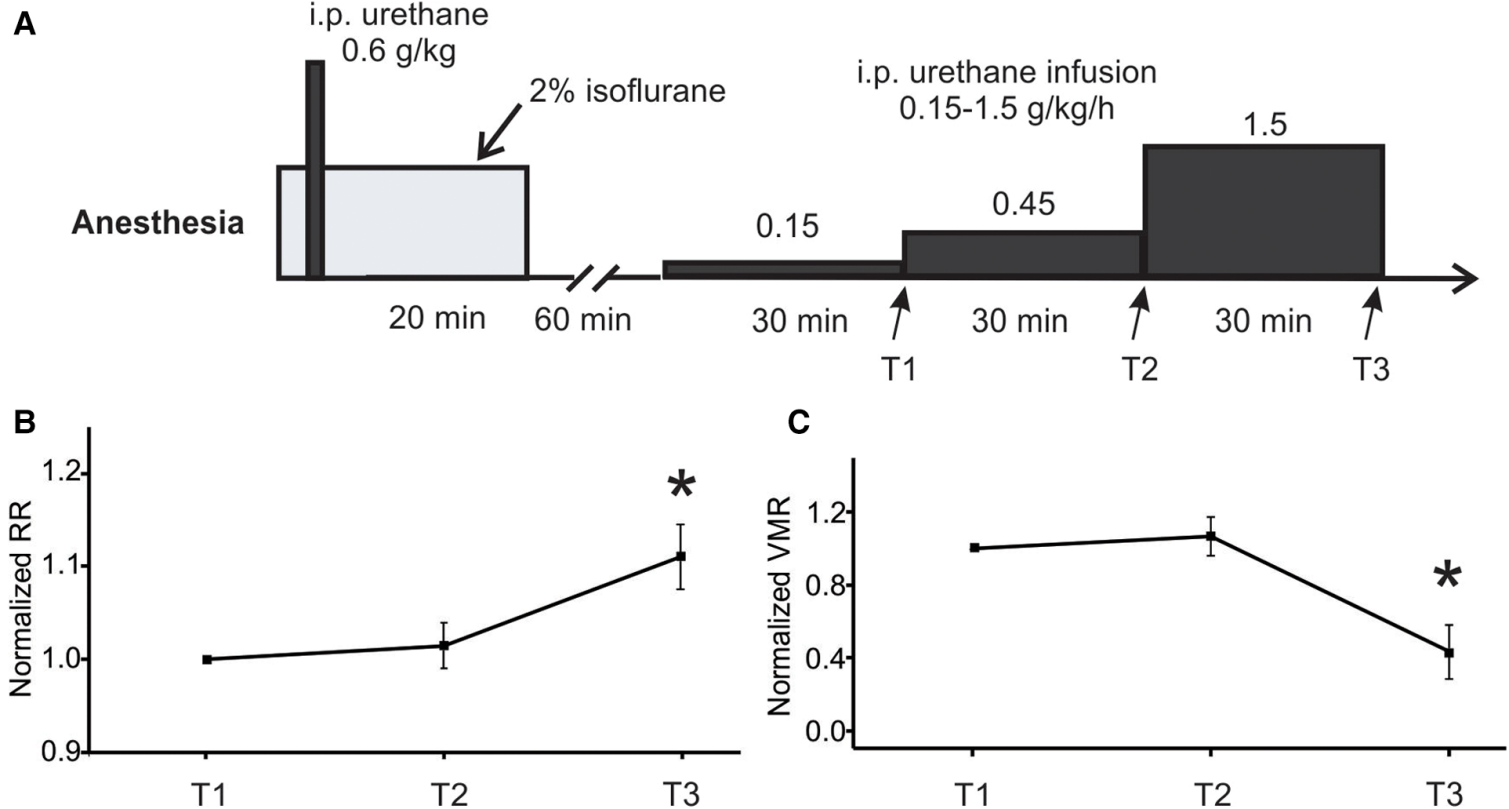
Optimization of low-dose urethane infusion rates by measuring RR and VMR. (**A**) The anesthesia protocol involved assessing three different urethane infusion rates in ascending order: 0.15, 0.45, and 1.5 g/kg/h. The RR and VMR were measured at the end of each infusion rate step indicated by T1, T2, and T3. (**B**) The RR and VMR were not significantly different between the infusion rates of 0.15 and 0.45 g/kg/h. However, the 1.5 g/kg/h infusion rate resulted in a significant increase in RR and a decrease in VMR when compared to the two lower infusion rates. * indicates *p* < 0.05 (*n* = 6). RR, respiratory rate; VMR, visceral motor responses.

The rate of low-dose urethane infusion is critical to maintain optimal mouse anesthesia, i.e., the absence of locomotion and any other volitional movement while the presence of spino-spinal reflex (e.g., plantar reflex) and spino-bulbo-spinal reflex (i.e., the VMR to CRD). Thus, three rates of low-dose urethane infusion (0.15, 0.45, and 1.5 g/kg/h) are objectively assessed not only by the VMR to CRD but also by the respiratory rate as measured from EMG activities of the latissimus dorsi musculature (L.D. musculature in Figure 1A). Each rate of low-dose infusion was applied for 30 min (shown in Figure 2).

### 2.3. The effect of axial location of the distending balloon on VMR

Since the distal ∼30 mm of the colorectum is differentially innervated by two distinct afferent pathways between the proximal colonic and distal rectal regions (26), the evoked VMR to CRD are likely different with distending balloon inserted in distal vs. proximal regions of the 30-mm-long colorectum. Thus, we systematically assessed the effect of axial location of the balloon in the colorectum on the evoked VMR by controlling the *balloon depth* in the colorectum, which is defined as distance between the distal end of the balloon and the anus. We control the balloon depth to be 0, 5, 10, 15, 20, 25, and 30 mm by marking and taping the connected catheter to the mouse tail. VMR to CRD were repeatedly recorded from the same animal with different balloon depths. At least 10 min was given between successive CRD.

### 2.4. VMR measurement in mice with TNBS-induced visceral hypersensitivity

Our prior report showed that intracolonic enema of TNBS induced persistent behavioral visceral hypersensitivity in male mice (11). In this study, we implemented the same mouse model to assess the effect of axial location of the distending balloon on VMR to CRD in both male and female mice. Mice were anesthetized by 2% isoflurane inhalation, transanally administered with TNBS (0.2 ml @ 10 mg/ml in 50% ethanol; Sigma-Aldrich, St. Louis, MO, United States) via a 22-gauge feeding needle (#18061-22, Fine Science Tools, Foster City, CA, United States), and held in a head down position (∼30°) for 5 min to preserve TNBS in the colorectum. Dietary gel (NGB-1, Bio-Serv, Flemington, NJ, United States) was provided to mice showing severe weight loss (>5% original body weight). Mice at 10–14 days following TNBS treatment were used in the current study, a time period as characterized by our prior study showing significant behavioral visceral hypersensitivity (11).

### 2.5. Analysis and statistics

EMG activities evoked by CRD were recorded from the abdominal oblique musculature, digitized at 2000 Hz, and processed off-line using customized MATLAB scripts. The EMG signals were rectified for calculating the area under the curve (AUC), which was used to evaluate the level of VMR to CRD (27). VMR evoked by CRD was quantified as the AUC values during the 5 s CRD subtracted by the AUC of the 5 s pre-distending baseline recording. Results are expressed as means ± standard error (SE). One-way ANOVA and Tukey's repeated *post-hoc* comparisons were performed as appropriate using SigmaStat v4.0 (Systat Software, San Jose, CA, United States). Differences were considered significant when *p* < 0.05.

## 3. Results

### 3.1. Determine the rate of low-dose urethane infusion to achieve consistent VMR to CRD

The main objective of the study is to establish a new anesthesia protocol for robust recordings of VMR to CRD in mice, the critical parameter of which is the rate of continuous infusion of low-dose urethane (Figure 1B). As shown in Figure 1A, VMR to CRD was recorded by the EMG electrode pair on the right E.O. musculature. RR was recorded by the EMG electrode pair on the L.D. musculature. The balloon depth in the colorectum was maintained at 5 mm in this study. In six mice (three females and three males), three ascending rates of continuous urethane infusion were assessed (0.15, 0.45, and 1.5 g/kg/h), each with a duration of 30 min (Figure 2A). The VMR and RR were recorded at the end of each 30-min duration as indicated by T1, T2, and T3 in Figure 2A. The VMR evoked by the 60-mmHg stepped pressure recorded at T1, T2, and T3 in each mouse were normalized by the VMR recorded at T1 with the lowest infusion rate (0.15 g/kg/h). As shown in Figure 2B, the infusion rate of 1.5 g/kg/h resulted in a significant increase in RR recorded at T3 (one-way ANOVA with repeated measure, *F*_2,15_ = 6.82, *p* < 0.05; *post-hoc* comparison, *p* = 0.015 vs. T2, *p* = 0.016 vs. T1). Also as shown in Figure 2C, the rate of 1.5 g/kg/h significantly suppresses the VMR to CRD as recorded at T3 (one-way ANOVA with repeated measure, *F*_2,15_ = 9.06, *p* < 0.5; *post-hoc* comparison, *p* = 0.004 vs. T2, *p* = 0.009 vs. T1). There was no significant difference in RR or VMR between the urethane infusion rates of 0.15 and 0.45 g/kg/h measured at T1 and T2, respectively. Thus, we maintained the rate of low-dose urethane infusion of no more than 0.3 g/kg/h for the rest of the study.

### 3.2. Comparison of VMR to CRD from different abdominal musculatures

**Figure 3:**
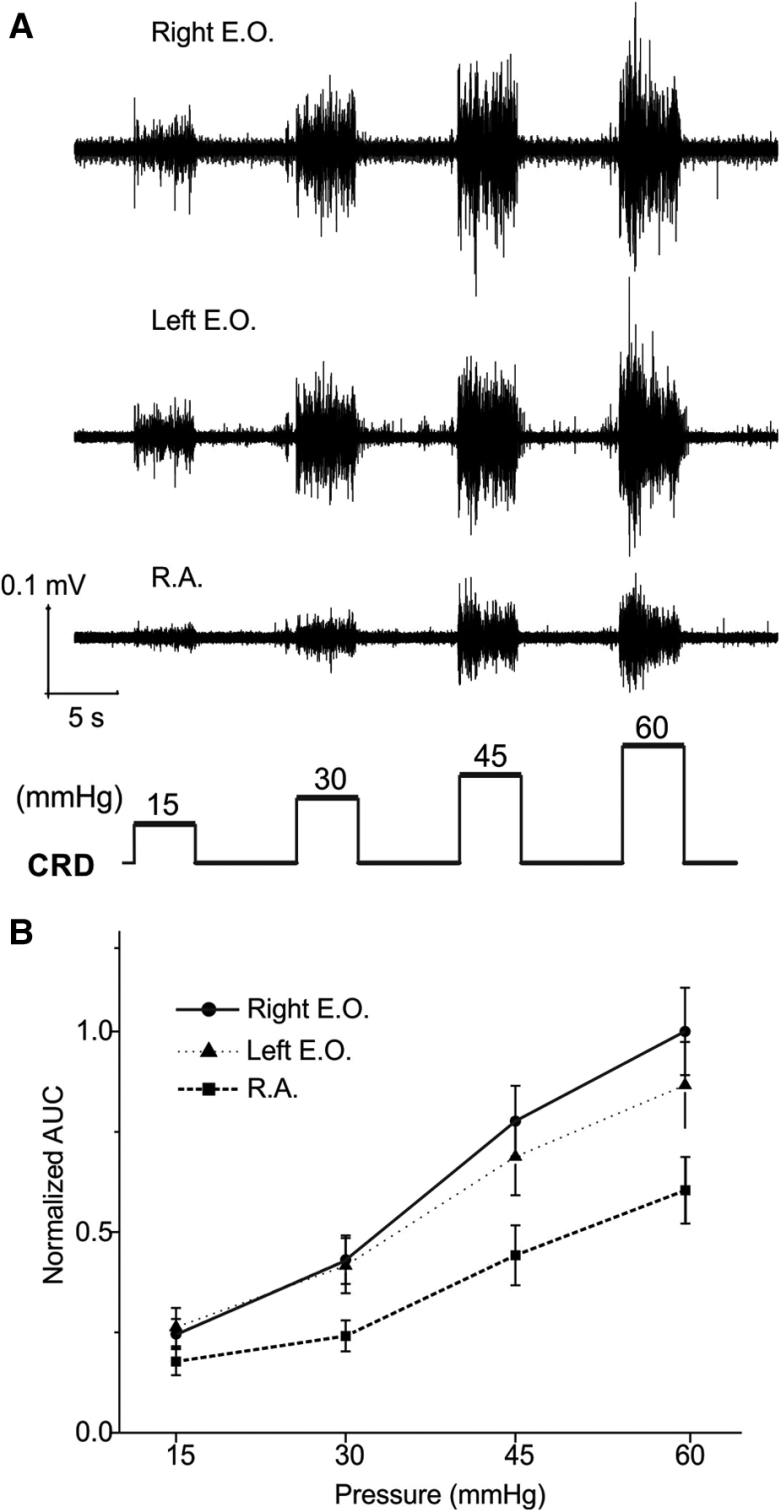
VMR to CRD recorded simultaneously from three different abdominal muscles: the left and right E.O. and R.A. muscles. (**A**) Representative EMG recordings of VMR from three abdominal muscles from one mouse in response to stepped CRD pressures: 15, 30, 45, and 60 mmHg. (**B**) Normalized AUC of EMG recordings from the three different abdominal muscles (*n* = 16). VMR, visceral motor responses; CRD, colorectal distension; EMG, electromyographic; AUC, area under the curve; E.O., external oblique; R.A., rectus abdominis.

The EMG magnitude as a metric of VMR is affected not only by the muscle recorded from but also the spacing between the two differential electrodes (∼1 mm). As shown in Figure 1A, we implanted three pairs of electrodes to simultaneously record from bilateral E.O. and the medial R.A. musculatures in 16 mice (8 females and 8 males). The balloon depth in the colorectum was set to be 5 mm in all recordings. The VMR recordings were processed as AUC values from four distending pressures (15, 30, 45, and 60 mmHg), which were normalized by the AUC value of 60 mmHg CRD (100%) recorded from the right E.O. Representative VMR from the right E.O., left E.O., and R.A. musculatures are displayed in Figure 3A, and the pooled data from 16 mice are displayed in Figure 3B. The VMR recorded from left and right E.O. musculatures were not significantly different (two-way ANOVA with repeated measure, *F*_2,510_ = 33.262, *p* < 0.0001; *post-hoc* comparison *p* = 0.19 for left vs. right E.O.), both of which are significantly higher than from the R.A. (*post-hoc* comparison, *p* < 0.0001 for right E.O. vs. R.A.; *p* < 0.0001 for left E.O. vs. R.A.). Thus, we only recorded VMR from the right E.O. for the rest of this study.

### 3.3. Assess repeatability of VMR recordings by repetitive CRD

**Figure 4:**
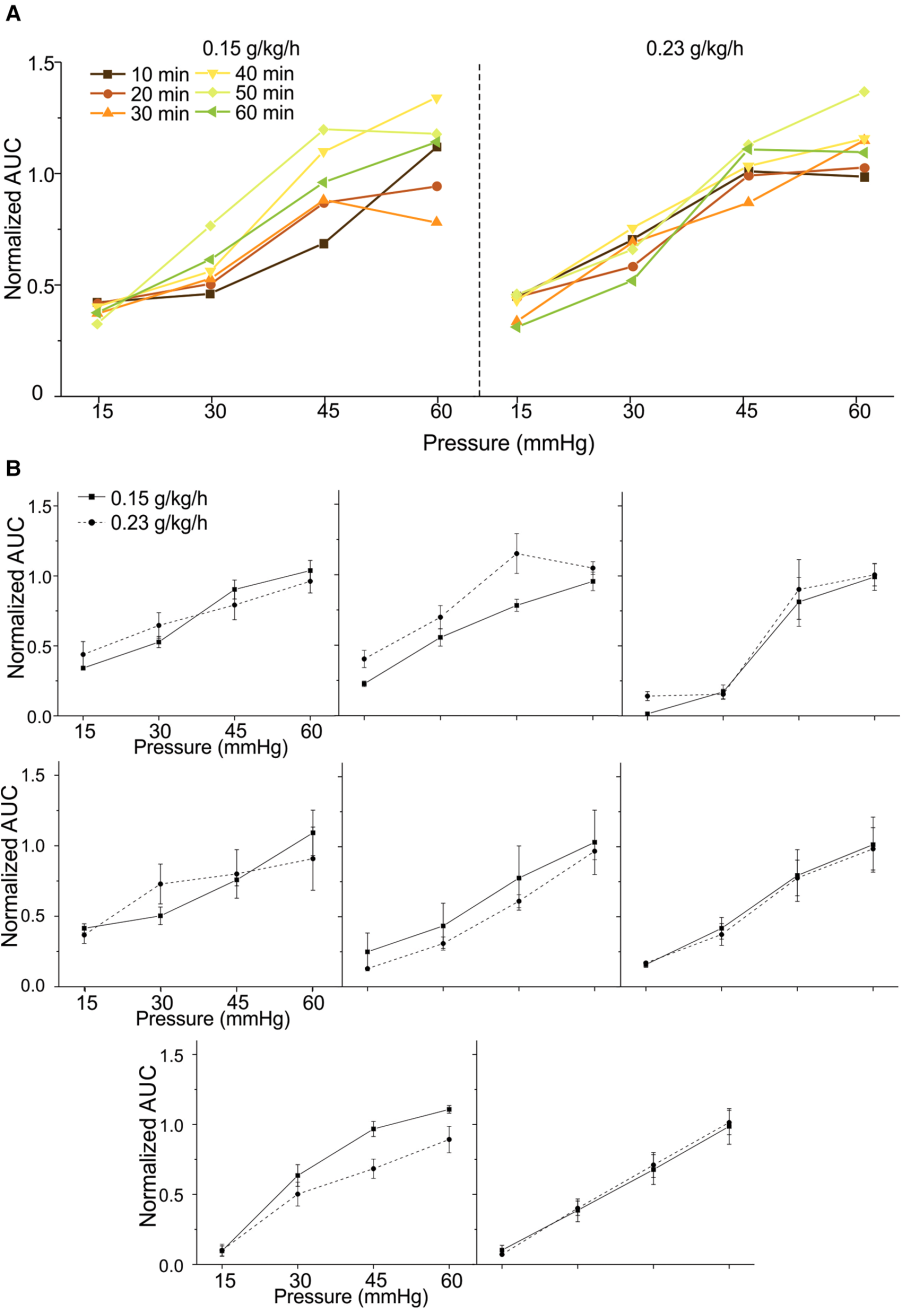
Repeated measurement of VMR to CRD in mice under the new urethane anesthesia protocol. (**A**) Representative VMR responses recorded 12 times from one mouse under 0.15 and 0.23 g/kg/h urethane infusion (six times each) within a 2-h period. The VMR is displayed as the normalized AUC of the EMG signals. (**B**) The average VMR to CRD recorded from eight mice under the 0.15 and 0.23 g/kg/h urethane infusion (six times each). The average VMR was recorded under the same infusion rate in each mouse. VMR, visceral motor responses; CRD, colorectal distension; EMG, electromyographic; AUC, area under the curve.

We aim to advance the VMR recordings from anesthetized mice as a reliable test bench to objectively assess various neuromodulatory interventions for treating visceral pain. Thus, we systematically determine the repeatability of VMR recordings evoked by multiple CRD within the 2-h time window during the low-dose urethane infusion in eight mice. From four mice (two females and two males), VMR to CRD was recorded six times during the first 60 min period at the low-dose urethane infusion rate of 0.15 g/kg/h and recorded for another six times during the second 60 min period at the infusion rate of 0.23 g/kg/h. The interval between successive CRD was maintained at 10 min throughout the test. Similar tests were conducted on another four mice (two females and two males) while switching the sequence of urethane doses, i.e., 0.23 g/kg/h of urethane in the first 60 min and 0.15 g/kg/h in the second 60 min period. Within each mouse, the AUC of all VMR was normalized by the response to the 60-mmHg distension in the first VMR. Figure 4A shows representative VMR recorded 12 times from one mouse at 0.15 g/kg/h and 0.23 g/kg/h urethane infusion. VMR recordings showed no within-sample differences in each of the eight mice recorded under the same rate of urethane infusion (two-way ANOVA with repeated measures, *F*_5,360_ = 1.925, *p* > 0.05 for all). In each of the eight mice, the VMR to CRD recorded at the same urethane infusion rates were averaged and plotted in Figure 4B, which showed no significant difference between 0.15 and 0.23 g/kg/h urethane in all of the eight mice tested (two-way ANOVA with repeated measures, *F*_1,56_ = 0.005, *p* > 0.05 for all). These results indicate that the new anesthesia protocol with low-dose urethane infusion (0.15–0.23 g/kg/h) enables robust and repeatable VMR recordings to CRD with 10 min intervals for at least 2 h.

### 3.4. CRD of proximal colonic region produces significantly lower level of VMR than of distal rectal region

**Figure 5:**
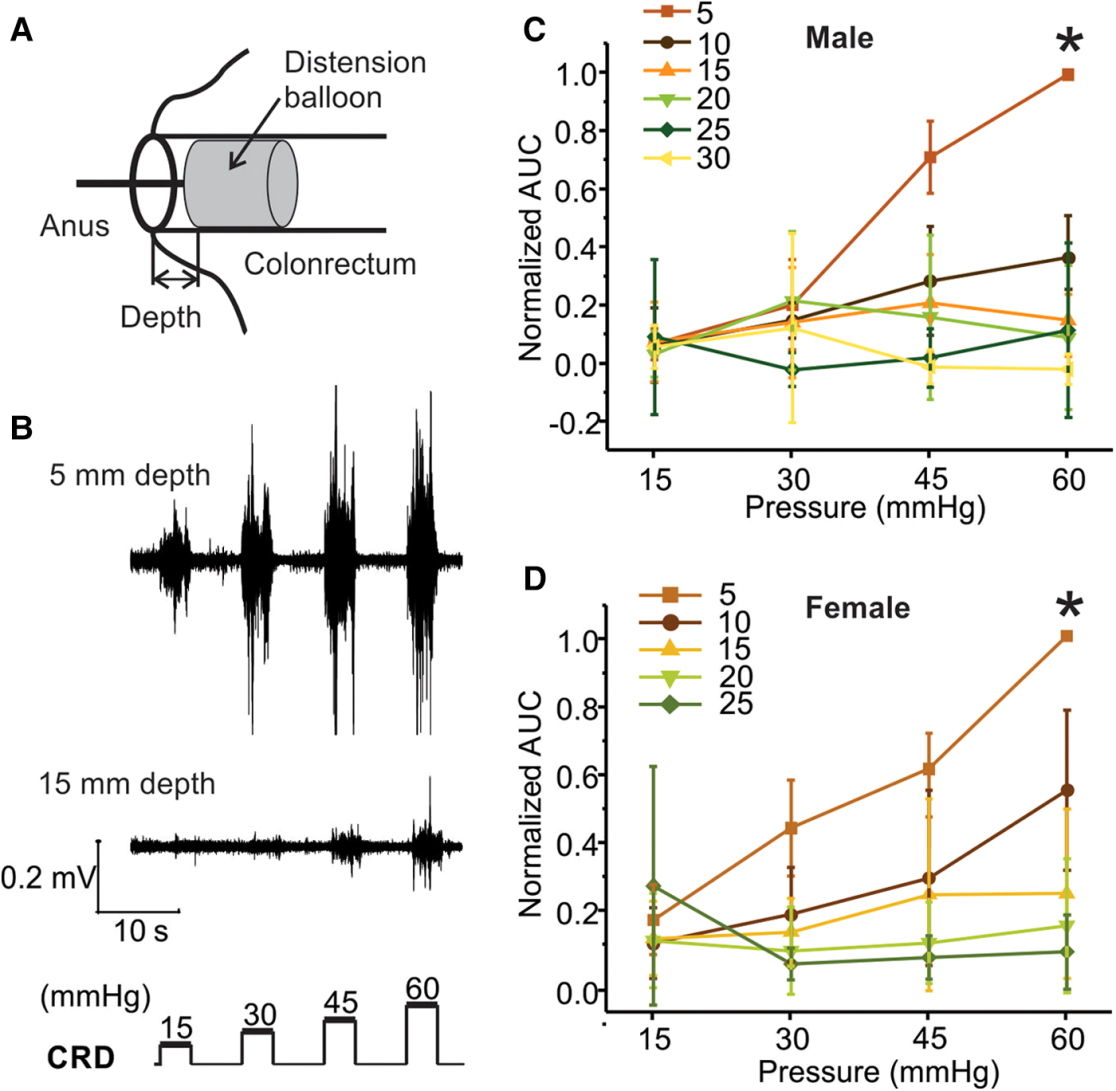
The effect of balloon depth in the colorectum on VMR to CRD. (**A**) Schematic representation of the precise control of balloon location in the colorectum by the balloon depth, i.e., the distance between the end of the balloon and the anus. (**B**) Representative EMG signals of VMR recorded from shallow (5 mm) and deep (15 mm) balloon depths from the same mouse. (**C**) Normalized VMR to CRD recorded from male mice at six different balloon depths: 5, 10, 15, 20, 25, and 30 mm. (**D**) Normalized VMR to CRD recorded from female mice at five different balloon depths: 5, 10, 15, 20, and 25 mm. * indicates *p* < 0.05 (*n* = 12). VMR, visceral motor responses; CRD, colorectal distension; EMG, electromyographic.

Within the same mouse, VMR to CRD was assessed with a distending balloon positioned at different depths in the colorectum as illustrated in Figure 5A. The representative VMR to CRD at balloon depth of 5 vs. 15 mm from the same mouse is displayed in Figure 5B, indicating the dramatic reduction of VMR with increased balloon depth in the colorectum. The VMR were recorded with balloon depths up to 30 mm into the anus in male mice and 25 mm in female mice. VMR from six male mice recorded at different balloon depths are normalized by the AUC evoked by the 60-mmHg pressure step with 5-mm balloon depth. Figures 5C,D show the average responses from six male and six female mice, respectively. VMR to CRD is significantly higher when recorded at the lowest balloon depth (5 mm) as compared to deeper distension with increased depth (10–20 mm) in both male (two-way ANOVA, *F*_3,560_ = 5.151, *p* < 0.05; *post-hoc* comparison, 5 mm vs. others, *p* < 0.05) and female mice (two-way ANOVA, *F*_3,560_ = 3.038, *p* < 0.05; *post-hoc* comparison, 5 mm vs. others, *p* < 0.05).

### 3.5. Intracolonic TNBS produced differential sensitization of VMR to CRD between male and female mice

**Figure 6:**
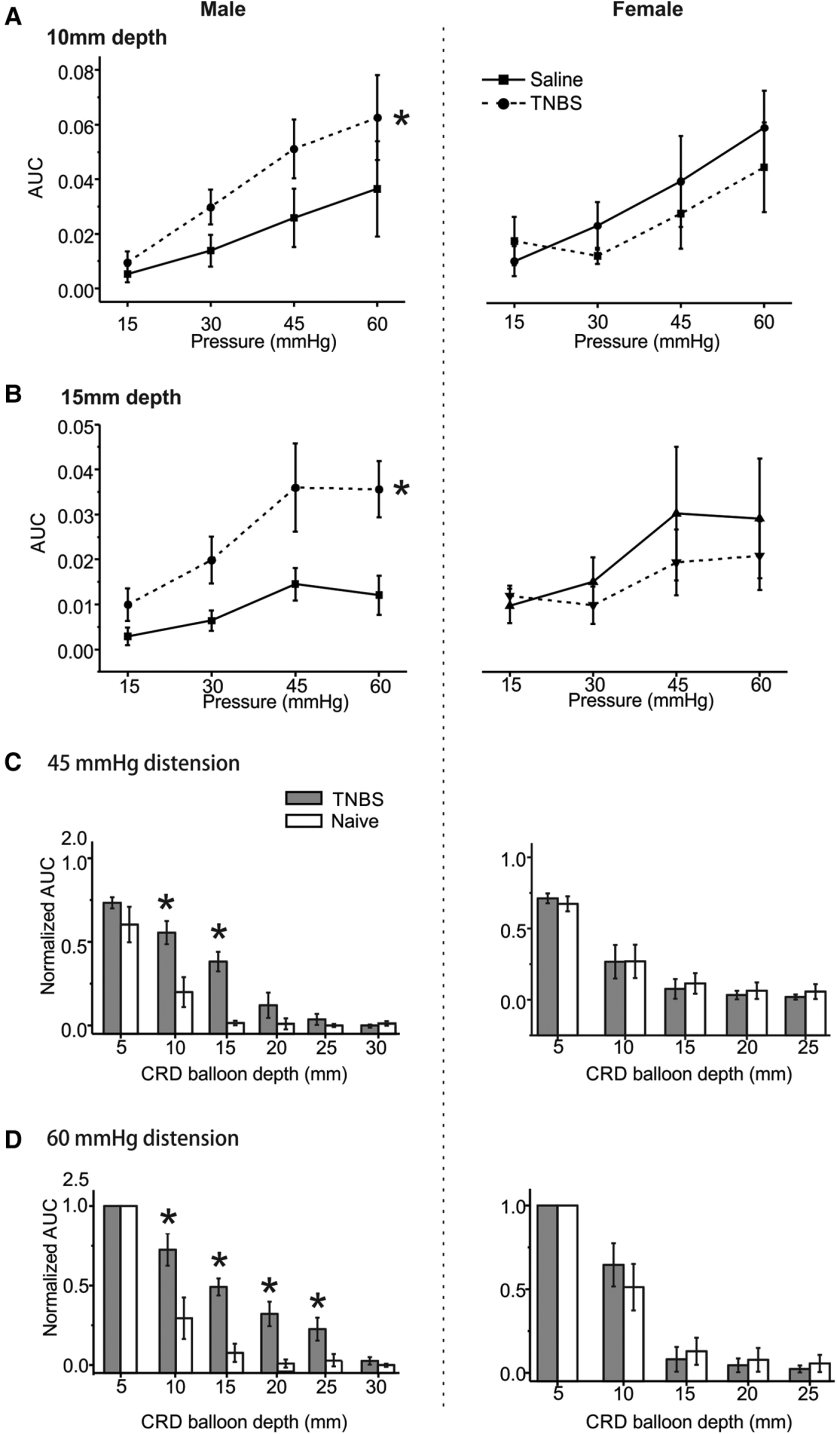
The effect of intracolonic TNBS on VMR to CRD in male and female mice. The visceral hypersensitivity following intracolonic TNBS and saline (as a control) was assessed in male and female mice by VMR to CRD at balloon depths of 10 mm (**A**) and 15 mm (**B**). (**C**) Normalized VMR to 45 mmHg stepped distension at different balloon depths. The VMR was normalized by the response to 60 mmHg distension step at 5 mm balloon depth. (**D**) Normalized VMR to 60 mmHg stepped distension at different balloon depths. * indicates *p* < 0.05 (*n* = 6). VMR, visceral motor responses; CRD, colorectal distension; TNBS, 2,4,6-trinitrobenzenesulfonic acid.

We previously reported that intracolonic TNBS treatment produced prolonged behavioral visceral hypersensitivity in male mice from 7 to 14 days post-treatment (11), which is evidenced by increased VMR to CRD recorded in conscious mice. VMR to CRD were recorded in mice under the newly developed urethane anesthesia protocol (0.3 g/kg/h urethane infusion) with multiple balloon depths into the colorectum in both male (5–30 mm depth) and female mice (5–25 mm depth). Tests were conducted in mice receiving intracolonic saline (control) or TNBS. As shown in Figure 6A for 10 mm balloon depth, the VMR to CRD is significantly higher in male mice receiving TNBS than those receiving saline (two-way ANOVA, *F*_1,40_ = 4.391, *p* < 0.05) but is not significantly different between female mice receiving TNBS and saline (two-way ANOVA, *F*_1,40_ = 0.62335, *p* = 0.434). This sex difference in TNBS-induced increase in VMR to CRD is also observed with 15 mm balloon depth as shown in Figure 6B, i.e., sensitized response by TNBS only in male mice (two-way ANOVA, *F*_1,40_ = 14.43158, *p* < 0.05) but not in female mice (*F*_1,40_ = 0.64, *p* = 0.428). VMR to CRD in saline-treated groups showed no significant sex differences for both 10 and 15 mm balloon depths (*F*_1, 40_ = 1.946, *p* > 0.05 for 10 mm, *F*_1, 40 _= 3.723, *p* > 0.05 for 15 mm). CRD recorded in each mouse were normalized by the response to 60 mmHg distension at 5 mm balloon depth. All responses to 45 mmHg distension at different balloon depths from saline and TNBS groups were pooled together and displayed in Figure 6C, and all responses to 60 mmHg displayed in Figure 6D. In male mice, VMR to distension of 45 mmHg was significantly higher at balloon depth of 10 and 15 mm in the TNBS group than in the saline group (Two-way ANOVA, F_1,20_ = 26.659, *p* < 0.05; *post-hoc* comparison), whereas female mice showed no difference between the TNBS and the saline groups at all tested balloon depths. Similarly, VMR to 60 mmHg distension were significantly increased in male mice in the TNBS group as compared to the saline group at balloon depths of 10, 15, 20, and 25 mm (two-way ANOVA, *F*_1,40_ = 36.421, *p* < 0.05; *post-hoc* comparison) while female mice showed comparable VMR between the TNBS and the saline groups.

**Figure 7:**
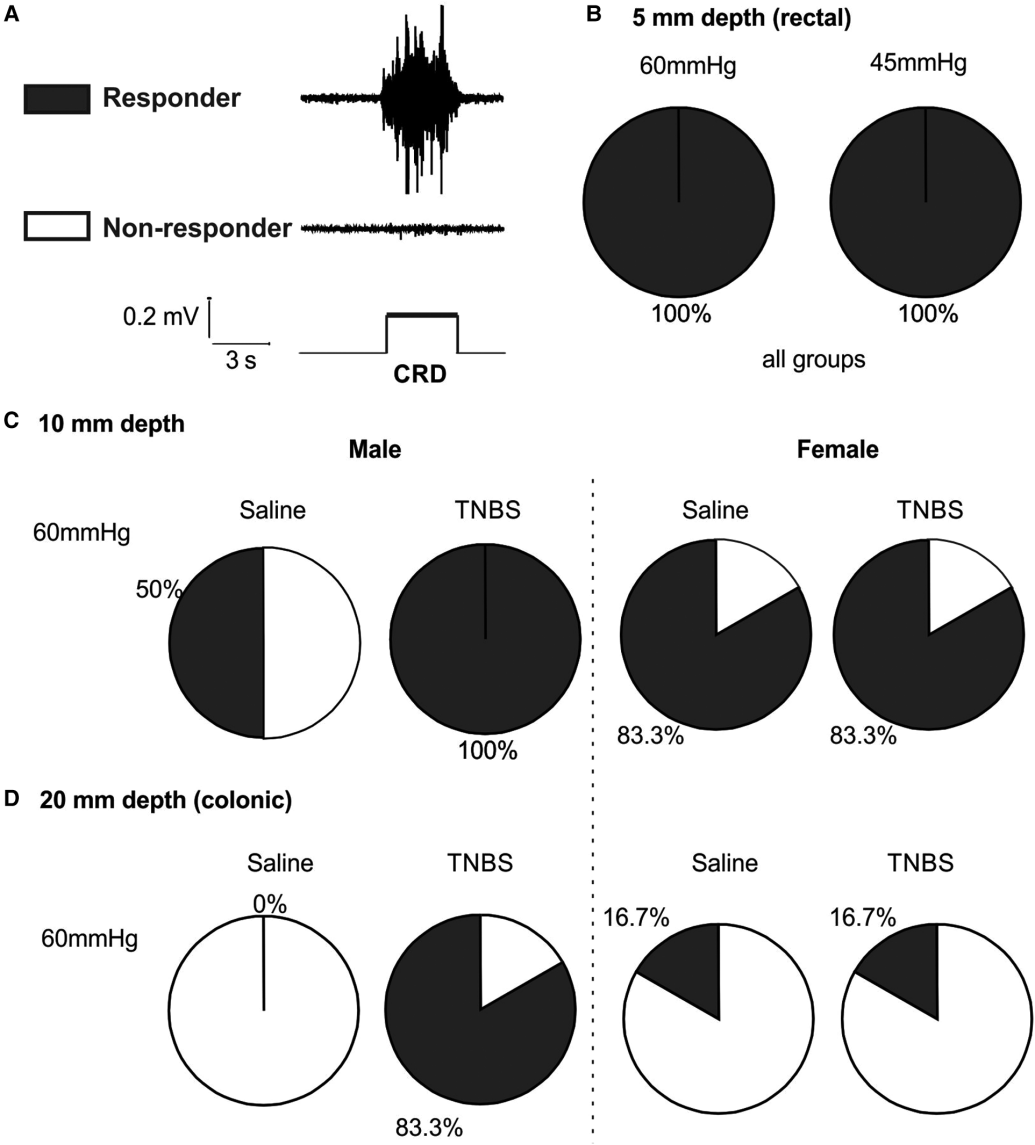
Intracolonic TNBS sensitizes the VMR to colonic distension in male mice. (**A**) Representative EMG recordings from a mouse with positive VMR responses to a distending pressure (responder) and from another mouse with negative VMR responses (non-responder). (**B**) Mice from all groups are responders to rectal distension of 45 and 60 mmHg with a shallow balloon depth of 5 mm. (**C**) Male mice receiving TNBS showed a higher responder proportion to 60 mmHg distension of 10 mm balloon depth than male mice receiving saline. Female mice showed no difference in the proportions of responders between the saline and TNBS groups. (**D**) Male mice receiving TNBS have sensitized responses to colonic distension (20 mm balloon depth) with 83.3% responders as compared to 0% responders in the saline group. Again, female mice showed no difference in the proportions of responders between the saline and TNBS groups. TNBS, 2,4,6-trinitrobenzenesulfonic acid; VMR, visceral motor responses; EMG, electromyographic.

We set the threshold for positive VMR response as five times the root mean square of the baseline noise level of the EMG signals. As illustrated in Figure 7A, mice with positive response to stepped distension are considered responders and those with negative response are non-responders. As shown in Figure 7B, mice of either sex receiving TNBS or saline are all responders to 45 and 60 mmHg distension at balloon depth of 5 mm. As summarized in Figure 7C for 10-mm balloon depth, only 50% mice are responders to 60 mmHg distension in male mice receiving saline, in comparison to 100% responders in male mice receiving TNBS. In contrast, female mice had comparable proportions of responders (83.3%) between the saline and the TNBS groups. At a balloon depth of 20 mm shown in Figure 7D, no male mice in the saline-treated group responded to 60 mmHg distension, whereas 83.3% in the TNBS-treated group did. Again, the proportions of responders in female mice were not different between saline (16.7%) and TNBS groups (16.7%).

## 4. Discussion

From the current study, we have systematically established an anesthesia protocol that enables the robust and repeatable measurement of VMR to CRD in anesthetized mice for at least 2 h. Increased abdominal muscle activity is considered a pseudoaffective reflex of visceral nociception that requires functioning spino-bulbo-spinal neural circuitry above the spinal level. Cervical spinal cord transection (C1) completely abolishes the VMR to the CRD (12). Many general anesthesia methods used in animal studies suppress supraspinal neural activities and thus significantly attenuate or completely inhibit the VMR to CRD, including steroidal anesthetic alphaxalone/alphadolone (12), isoflurane (28), halothane (29), ketamine (30), and alpha-chloralose (12). Pentobarbital sodium, previously used as a sedative and preanesthetic in patients, preserves the VMR to CRD in mice and was used by several prior studies [e.g., (31)]. However, its discontinued manufacturing and heightened regulatory control in the United States have prevented its wider application in recent laboratory research. General anesthesia by a single i.p. injection of urethane (typically 1–1.5 g/kg) was widely used to preserve the VMR to CRD in anesthetized rats [e.g., (32–35)]. In comparison, a similar urethane anesthesia protocol was only implemented by a handful of mouse studies for recording VMR to CRD under anesthesia [i.e., (22, 25)], suggesting the need to further improve and optimize the urethane anesthesia protocol in mice.

In the current study, we demonstrated that continuous i.p. infusion of a low-dose urethane enables a consistent anesthesia condition for repeated assessment of VMR to CRD. To initiate anesthesia, we used an initial bout of 0.6 g/kg urethane infusion combined with continuous isoflurane inhalation (2%), which not only removes voluntary locomotion of the mouse but also inhibits the spino-spinal reflex from forceps pinching of the hind paw. Consistent with the inhibitory effect of isoflurane reported previously (28), we did not record any VMR to CRD during and within 30 min after terminating the isoflurane anesthesia. We then narrowed down the range of urethane dose for continuous infusion by precisely measuring the respiratory rate, which closely correlates with the level of brain activity during anesthesia and sleep (36). Different doses of urethane anesthesia reportedly recapitulate distinct stages of natural sleep in rodents: slow-wave sleep (SWS) at low-dose and rapid eye movement (REM) sleep at high dose with a respiratory rate higher in the REM phase than in the SWS phase (36). We observed that the respiratory rate at 0.15 and 0.45 g/kg/h urethane infusion is comparable but significantly lower than at 1.5 g/kg/h, suggesting the SWS phase at the dose of 0.15–0.45 g/kg/h and REM phase at 1.5 g/kg/h. VMR to CRD was absent at an infusion rate of 1.5 g/kg/h, consistent with the prior finding that high dose of urethane completely abolished the VMR in rats (12). Since the SWS sleep phase features stable brain neural activities and autonomic signals, we limited the urethane infusion rate to below 0.45 g/kg/h. The lower range of the perfusion rate is justified by the stable respiratory rate in the absence of voluntary and involuntary movement, indicative of sufficient anesthesia. Remarkably, we demonstrated that six consecutive measurements of VMR to CRD with 10 min intervals are comparable with no significant difference in each of the eight tested mice at two perfusion rates of urethane: 0.15 and 0.23 g/kg/h. In addition, the mean VMR to CRD recorded under 0.15 and 0.23 g/kg/h urethane infusion is comparable within each mouse. These systematic studies strongly indicate that i.p. infusion of 0.15–0.23 g/kg/h urethane provides an optimal anesthesia condition to allow repeatable measurement of VMR to CRD for at least 2 h.

We limited the age of the mice to 8–12 weeks when optimizing the urethane anesthesia condition. We are fully aware that age is a significant factor impacting the level of anesthesia. Older mice have a slower metabolism rate (37), which likely contributes to a longer time to achieve anesthesia and longer carryover period after terminating the anesthesia. From our unpublished observation, after the initial bout of urethane and isoflurane anesthesia, aged mice (>20 weeks of age) require a longer isoflurane clearing time to show VMR to CRD than younger adult mice (8–12 weeks). The anesthesia conditions established from the current study will be an ideal baseline for further optimization in studies using mice at other age ranges.

Using the anesthetized mouse model, we systematically assessed two key factors that affect the quantitative measurement of VMR to CRD via EMG recordings, i.e., EMG electrode location in different muscles and balloon depth in the colorectum. The visceromotor reflex to visceral nociception evokes higher activities in certain skeletal or smooth muscles than others. For example, noxious stomach distension evokes the highest level of EMG signals in skeletal muscles in the neck rather than back muscles or abdominal smooth muscles; within the neck muscles, the signal is higher in the acromiotrapezius muscle than in the sternomastoideus muscle (38). Through simultaneous EMG recordings from three different abdominal smooth muscles (left and right E.O. and R.A. musculatures), we showed that VMR to CRD evoked a higher EMG magnitude in E.O. than in the R.A. musculature. Remarkably, the recorded EMG signals from the left and right E.O. showed no statistical difference despite being recorded by different pairs of electrodes that would likely have different input impedance due to different spacing between the two bipolar inputs. This suggests that EMG signals are a reliable metric of evoked E.O. muscle activities, not significantly impacted by the electrode configurations, and can be implemented to compare the level of VMR between different animals. The distending balloon location in the colorectum, as indicated by the balloon depth, significantly impacts the evoked EMG magnitude: increasing the balloon depth from 5 to 15 mm resulted in about a 60%–70% reduction in EMG magnitude. The mouse distal colorectum consists of colonic and rectal regions that are predominantly innervated by the lumbar splanchnic nerve (LSN) and pelvic nerve (PN) pathways, respectively (26, 39). Given the balloon length of 15 mm, CRD of 5 mm depth will likely stimulate both LSN and PN afferents, whereas CRD of 15 mm will stimulate mostly the LSN afferents. Our result is consistent with a prior nerve transection study indicating the dominant role of the PN pathway in driving the VMR to CRD; transecting the LSN does not significantly affect the magnitude of VMR, whereas transecting the PN completely abolishes the VMR (31). Taken together, the current results indicate that consistent EMG recordings of VMR to CRD will require (1) recording electrodes to be on the E.O. musculature and (2) distending balloons to be positioned in the rectal region, preferably 5–10 mm into the anus.

Under the new urethane anesthesia protocol, we demonstrated that the VMR to CRD was significantly higher in male mice 10–14 days after TNBS treatment than in male mice receiving saline as a control, consistent with prior findings of VMR recorded in conscious male mice before and after TNBS treatment (11). However, VMR recorded in urethane-anesthetized mice can only be recorded at one time point because mice cannot survive under urethane anesthesia. As discussed earlier, VMR quantified by EMG activities is not significantly affected by electrode configurations, enabling a comparison of VMR to CRD between the TNBS and saline groups. The difference in VMR between the saline and TNBS groups of male mice is apparent only when the balloon predominantly distends the colonic region (i.e., 10 and 15 mm balloon depth). In contrast, no difference in VMR was observed with a 5 mm balloon depth that predominantly distends the rectal region. This is consistent with the finding of normalized VMR recorded at different balloon depths from the same mouse, which shows significantly higher VMR in the TNBS group at balloon depths of 10–25 mm than in the saline group. Interestingly, there is a significant sex difference in TNBS-induced visceral hypersensitivity. There are no significant differences in VMR to CRD in female mice between the TNBS- and saline-treated groups. Additionally, the normalized VMR to CRD showed no difference between the TNBS and saline groups at all balloon depths. A prior study indicates that VMR to CRD in female rats is significantly affected by the estrous cycle, higher in proestrus and estrus phases than in diestrus and metestrus phases (28). We did not track the cycle of female mice in the current study. Thus, the lack of a significant difference in the current study may be due to cycle-induced variations of VMR. Nonetheless, the normalized VMR recorded in each female mouse, which accounts for the between-sample variations, also showed no apparent sensitization of VMR to CRD in female mice receiving TNBS compared to female mice receiving saline. Thus, intracolonic TNBS does not induce significant visceral hypersensitivity in female mice, similar to our recent study showing comparable VMR to CRD between intracolonic saline and zymosan-treated female mice (40). This sex difference in visceral hypersensitivity induced by colonic insult *per se* can be attributed to the decreased colonic epithelial permeability in female mice due to the upregulation of the tight junction proteins occludin and junctional adhesion molecule (JAM)-A via the sex hormone estrogen (41–43). The underlying mechanisms of sex hormone-mediated tight junction function of intestinal epithelia in visceral hypersensitivity await further study.

## 5. Conclusion

We developed a urethane anesthesia protocol for measuring the VMR to CRD in mice. The protocol involves an initial urethane dose (0.6 g/kg) followed by a low-dose infusion (0.15–0.23 g/kg/h) for up to 2 h. Repeated CRD assessments showed a consistent VMR magnitude. VMR recorded from the E.O. muscles was higher than from the R.A. muscles. Additionally, rectal distension with shallower balloon depth produced a significantly higher VMR magnitude than colonic distension with deeper balloon depth. We investigated the effects of intracolonic TNBS treatment on VMR to CRD in male and female mice. TNBS treatment induced significant visceral hypersensitivity in male mice at 10–14 days post-treatment, as evidenced by higher VMR magnitude. Female mice showed comparable VMR between the TNBS and saline groups. Further studies are required to understand the underlying neuronal and hormonal mechanisms for the apparent sex difference in TNBS-induced visceral hypersensitivity. Our findings provide a reliable methodology for studying CRD-induced VMR in mice and contribute to the understanding of sex differences in visceral hypersensitivity.

## Data Availability

The raw data supporting the conclusions of this article will be made available by the authors, without undue reservation.
